# Advances in the Diagnosis and Treatment of Follicular Thyroid Carcinoma: A Comprehensive Review

**DOI:** 10.7759/cureus.66186

**Published:** 2024-08-05

**Authors:** Vasundara Gopalan, Swati G Deshpande, Anup A Zade, Darshana Tote, Rahul Rajendran, Shubham Durge, Abhilasha Bhargava

**Affiliations:** 1 General Surgery, Jawaharlal Nehru Medical College, Datta Meghe Institute of Higher Education and Research, Wardha, IND

**Keywords:** thyroid cancer management, targeted therapies, molecular diagnostics, treatment strategies, diagnostic advances, follicular thyroid carcinoma

## Abstract

Follicular thyroid carcinoma (FTC) is the second most common type of thyroid cancer, presenting unique diagnostic and therapeutic challenges. This review provides a comprehensive analysis of the recent advancements in the diagnosis and treatment of FTC, emphasizing the significance of these developments in improving patient outcomes. We discuss the evolution of diagnostic techniques, including advancements in imaging modalities, fine needle aspiration biopsy, and molecular diagnostics, which have enhanced the accuracy of FTC detection and differentiation from benign conditions. The review also evaluates current treatment strategies, including surgical interventions, radioactive iodine therapy, and targeted therapies, examining their effectiveness and impact on patient prognosis. Additionally, we address ongoing challenges in FTC management, such as variability in treatment guidelines and disparities in care. Finally, the review explores emerging therapies and future research directions, highlighting innovations that may further optimize FTC management. By synthesizing current knowledge and identifying future research opportunities, this review aims to contribute to refining diagnostic and therapeutic approaches for FTC.

## Introduction and background

Thyroid carcinoma represents the most common endocrine malignancy, encompassing a range of histological subtypes with varying clinical behaviors [1]. The primary types include papillary thyroid carcinoma (PTC), follicular thyroid carcinoma (FTC), medullary thyroid carcinoma (MTC), and anaplastic thyroid carcinoma (ATC). Among these, FTC is the second most prevalent, accounting for approximately 10-15% of thyroid cancer cases [2]. Originating from the thyroid gland's follicular cells, FTC is distinguished by its unique biological characteristics and clinical management challenges. The global increase in thyroid cancer incidence is partly attributed to advancements in imaging technologies and heightened awareness, which have improved detection rates [3].

Studying FTC is crucial due to its distinct diagnostic and therapeutic challenges. Unlike PTC, which often presents with more recognizable histological features, FTC can be more challenging to differentiate from benign follicular lesions based solely on histological examination [4]. This difficulty in diagnosis can lead to delays in appropriate treatment. Furthermore, FTC is known for its tendency to invade blood vessels and potentially metastasize to distant organs, complicating its management compared to other types of thyroid cancer. Understanding FTC is vital for developing more effective diagnostic and treatment strategies, ultimately enhancing patient outcomes [5].

This review aims to provide a comprehensive overview of the recent advancements in the diagnosis and treatment of FTC. It will summarize the latest developments in diagnostic techniques, including imaging modalities, biopsy methods, and molecular diagnostics, which have improved the accuracy of FTC diagnosis. It will also review current treatment strategies, such as surgical options, radioactive iodine (RAI) therapy, and emerging targeted therapies, analyzing their effectiveness and outcomes. Additionally, the review will address the challenges and controversies in FTC management, including inconsistencies in treatment guidelines and disparities in patient care. Finally, it will explore future directions, highlighting ongoing research, new therapeutic approaches, and potential areas for further investigation. By addressing these objectives, this review aims to enhance understanding and improve the management of FTC.

## Review

Epidemiology and risk factors

Incidence and Prevalence of FTC

FTC is the second most prevalent form of thyroid cancer, accounting for approximately 10-20% of all thyroid neoplasms [6]. The incidence of FTC varies markedly depending on geographical location and dietary iodine levels. In the United States, between 1980 and 2009, the annual incidence of FTC was reported to be 11.9 cases per million women and 5.5 cases per million men [7]. As of 2022, FTC is estimated to represent about 10-15% of all thyroid cancer diagnoses in the U.S. Globally, the incidence can reach up to 40% in regions with iodine deficiency, highlighting the impact of dietary factors on thyroid cancer rates. The pooled global incidence of FTC is approximately 0.07 cases per 100,000 people [8].

Risk Factors for FTC

Risk factors for FTC can be classified into genetic, environmental, and hormonal categories. Genetic factors include specific mutations, particularly in the RAS oncogene, which have been linked to the development of FTC [9]. Familial syndromes, such as multiple endocrine neoplasia (MEN), can also increase the risk of developing thyroid cancers, including FTC. Environmental factors are also significant; iodine deficiency is strongly associated with thyroid carcinoma, with higher FTC rates reported in regions with endemic goiter [10]. Ionizing radiation, especially exposure during childhood from medical treatments or environmental disasters, is another well-established risk factor [11]. Hormonal influences contribute to the risk profile of FTC as well. The carcinoma is more commonly observed in women, with a female-to-male ratio of approximately 3:1. This suggests that hormonal changes related to menstruation and menopause may play a role, although the precise mechanisms remain unclear. These genetic, environmental, and hormonal factors collectively create a complex risk landscape for FTC [12].

Differences From Other Thyroid Cancers

FTC exhibits distinct characteristics that set it apart from other types of thyroid cancer, particularly PTC. One key difference lies in the demographic profile: FTC typically affects older individuals, generally between 40 and 60, whereas PTC is more common in younger patients. The median age at diagnosis for FTC is around 51 years, highlighting this age disparity [13]. Regarding metastatic behavior, FTC is known for its propensity to metastasize hematogenously rather than through lymphatic spread, which is more characteristic of PTC. Approximately 20% of FTC patients may present with distant metastases at diagnosis, whereas only 5-10% have lymph node metastases [14]. Additionally, FTC is classified into minimally invasive (MI-FTC) and widely invasive (WI-FTC) types based on the extent of capsular and vascular invasion. This classification can significantly impact treatment options and prognosis [15].

Pathophysiology and classification

FTC is distinguished by follicular cell differentiation, lacking the nuclear features typical of PTC. Diagnosing FTC necessitates histological evidence of capsular or vascular invasion, which can only be confirmed following complete surgical resection of the tumor. MI-FTC is characterized by capsular invasion alone or limited vascular invasion (fewer than four vessels). In contrast, WI-FTC exhibits extensive invasion into the thyroid and extrathyroidal soft tissue [6]. The molecular pathogenesis of FTC is believed to begin with point mutations that lead to dysregulation of the phosphatidylinositol-3-kinase (PI3K)/AKT signaling pathway. This dysregulation is often triggered by activating mutations in RAS, phosphatidylinositol-4,5-bisphosphate 3-kinase catalytic subunit alpha (PIK3CA), AKT1, or inactivating phosphatase and tensin homolog (PTEN). RAS oncogene mutations are prevalent in FTC, particularly in poorly differentiated (55%) and anaplastic carcinomas (52%) [16]. NRAS mutations have been reported in 17-57% of FTC cases. Additionally, the paired box 8 (PAX8)/peroxisome proliferator-activated receptor gamma (PPARG) gene fusion, resulting in a PAX8-PPARγ fusion protein, is identified in approximately one-third of FTC cases. Activating mutations in telomerase reverse transcriptase (TERT), which encodes TERT, are associated with the most severe clinical features and poorest prognosis in FTC [17]. The TNM staging system is utilized to classify differentiated thyroid cancer (DTC), with T describing the tumor size, N indicating lymph node involvement, and M denoting distant metastasis [18]. The World Health Organization (WHO) categorizes FTC into three types: MI-FTC, WI-FTC, and encapsulated angioinvasive. In summary, FTC diagnosis depends on histological evidence of capsular or vascular invasion, and it is characterized by genetic alterations that disrupt the PI3K/AKT pathway. The WHO classification and TNM staging system are essential for guiding treatment and predicting prognosis in FTC patients [18].

Diagnosis

FTC often presents asymptomatically in its early stages, making early detection difficult. Many cases are discovered incidentally during evaluations for unrelated issues. When symptoms do occur, they typically include a palpable neck lump, which is the most common presentation [19]. Patients may also experience vocal changes, such as hoarseness or alterations in voice, especially if the tumor invades surrounding structures. Additionally, difficulty swallowing may arise if the tumor compresses the esophagus. In more advanced stages, systemic symptoms like weight loss, fatigue, or pain in other body areas may indicate metastatic disease [19]. Several imaging techniques evaluate suspected FTC, with ultrasound being the primary modality. Ultrasound helps identify characteristics of thyroid nodules, such as size, echogenicity, and microcalcifications or irregular margins, which may suggest malignancy [20]. Computed tomography (CT) scans are employed to assess the extent of the disease, particularly in cases with suspected extrathyroidal extension or lymph node involvement. Magnetic resonance imaging (MRI) may be utilized in specific scenarios, especially for evaluating soft tissue involvement or when CT is contraindicated. Additionally, positron emission tomography (PET) scans can be valuable for assessing metastatic disease, particularly in recurrent or advanced FTC cases [20]. CT scans are employed to assess the extent of the disease, particularly in cases with suspected extrathyroidal extension or lymph node involvement. MRI may be utilized in specific scenarios, especially for evaluating soft tissue involvement or when CT is contraindicated. Additionally, PET scans can be valuable for assessing metastatic disease, particularly in recurrent or advanced FTC cases [20]. Fine needle aspiration biopsy (FNAB) is crucial for diagnosing FTC. This minimally invasive technique involves inserting a thin needle into the thyroid nodule to obtain a sample of cells for cytological examination [21]. FNAB is essential for distinguishing between benign and malignant nodules, although diagnosing FTC can be challenging due to its well-differentiated nature, which may resemble benign conditions. The Bethesda System for Reporting Thyroid Cytopathology is commonly used to classify FNAB results, providing a standardized approach that guides further management based on malignancy risk [21]. Molecular diagnostics have become increasingly important in evaluating FTC, offering additional insights that can influence treatment decisions [22]. Genetic testing for specific mutations, such as BRAF and RAS, helps assess the risk of malignancy and tailor management strategies. Gene expression profiling, which analyzes the expression of multiple genes, provides information about the tumor's behavior and potential aggressiveness. These tests assist in differentiating FTC from benign lesions and other types of thyroid cancer, enhancing diagnostic precision and informing therapeutic approaches [22].

Treatment options

Surgical Management

The surgical management of FTC primarily involves deciding between total thyroidectomy and lobectomy, influenced by factors such as tumor size, histological characteristics, and the presence of metastasis [23]. Total thyroidectomy, which involves the complete removal of the thyroid gland, is generally recommended for tumors larger than 1 cm, those with extrathyroidal extension, or cases with distant metastases. This approach is associated with a lower risk of recurrence and better long-term survival outcomes for patients with high-risk features [23]. Conversely, lobectomy, which entails removing only one lobe of the thyroid, may be appropriate for smaller tumors, especially those less than 1 cm without evidence of invasion or metastasis. Recent studies suggest that for tumors between 1 and 4 cm, survival rates following total thyroidectomy and lobectomy are comparable when adjusted for various risk factors [24]. This has led to a more conservative approach in select cases, particularly for patients with MI-FTC, who often have a favorable prognosis and may not require extensive surgical intervention. In pediatric patients, lobectomy is often preferred due to the lower risk of aggressive disease and the desire to preserve thyroid function [24]. In addition to the primary surgical procedure, lymph node dissection is crucial, especially when there is evidence of lymph node involvement [25]. Therapeutic neck dissection is recommended for patients with clinically apparent or biopsy-proven metastatic lymph nodes. Research indicates that many patients with MI-FTC do not have lymph node metastases, suggesting that surgery may be less necessary for this subgroup. The surgical approach for FTC should be individualized, considering tumor characteristics, patient age, and overall health to optimize outcomes and minimize complications [25].

RAI Therapy

RAI therapy is a vital treatment for DTC, especially for FTC. It is primarily indicated for patients who have undergone total thyroidectomy, as it helps ablate any remaining thyroid tissue and address residual disease [26]. RAI is particularly beneficial for high-risk patients, including those with aggressive tumor histologies, extensive lymph node involvement, or distant metastases. Additionally, RAI may be considered for patients with elevated serum thyroglobulin (Tg) levels post-surgery, even in the absence of structural disease, to prevent recurrence [26]. The effectiveness of RAI therapy is well-documented, showing significant improvements in disease-free and overall survival, particularly among high-risk populations. Research demonstrates that RAI effectively reduces the risk of structural recurrence in DTC patients, leading to better clinical outcomes. Typically, a single fixed dose of RAI suffices for many patients, achieving remission in a substantial percentage within a year following treatment. This effectiveness highlights RAI's role as a cornerstone in FTC management [27]. RAI works through several mechanisms. The primary mechanism involves the selective uptake of iodine by thyroid cells, including malignant ones, which preferentially absorb RAI (I-131). This selective uptake allows localized radiation to destroy cancerous cells. The emitted beta particles cause cellular damage and apoptosis in thyroid cells, effectively reducing tumor burden and preventing recurrence. This targeted approach maximizes therapeutic effects while minimizing damage to surrounding tissues [28]. While RAI therapy is generally well-tolerated, it can have several side effects. Hypothyroidism is one of the most common outcomes, requiring lifelong thyroid hormone replacement therapy. Patients may also experience sialadenitis, an inflammation of the salivary glands due to iodine uptake, leading to discomfort and swelling. Although the doses used in RAI therapy are considered safe, there is a potential risk of secondary malignancies with higher cumulative doses over time. Additionally, some patients may experience nausea and vomiting following treatment [29].

Targeted Therapies and Systemic Treatments

**Figure 1:**
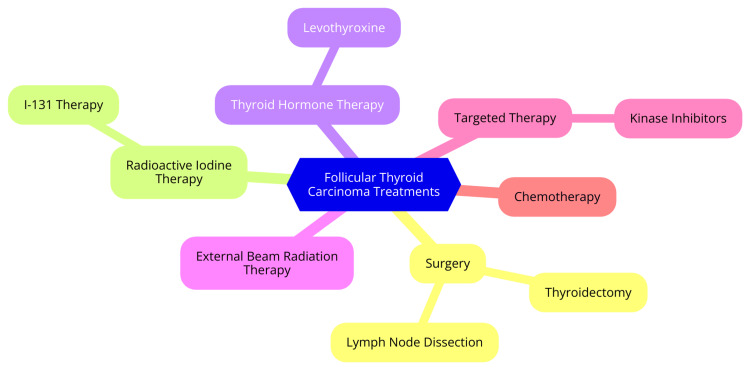
Treatment options for follicular thyroid carcinoma Image credit: Dr. Vasundara Gopalan

Targeted therapies and systemic treatments for FTC are increasingly significant, especially for patients with RAI-refractory disease. Tyrosine kinase inhibitors (TKIs), like lenvatinib and sorafenib, are FDA-approved for advanced thyroid cancers, including FTC. These multi-kinase inhibitors target critical pathways involved in tumor growth and angiogenesis, such as the vascular endothelial growth factor (VEGFR) receptor and platelet-derived growth factor (PDGF) receptor [30]. Lenvatinib has demonstrated notable efficacy in clinical trials, with a progression-free survival (PFS) of 18.3 months compared to 3.6 months for placebo. Sorafenib has also shown effectiveness, achieving a PFS of 10.8 months in trials, highlighting its potential in managing locally advanced or metastatic DTC [30]. TKIs work by inhibiting multiple receptor tyrosine kinases, crucial for cancer cell proliferation and survival. Despite their promise, challenges such as acquired resistance and adverse side effects persist, impacting treatment tolerability and overall survival rates [30]. Combination therapies are being explored to enhance the effectiveness of TKIs and overcome resistance mechanisms. For example, combining TKIs with heat-shock protein 90 (HSP90) inhibitors, such as onalespib, is a strategy to address resistance. HSP90 inhibitors target molecular chaperones that assist in the proper folding of proteins involved in cancer progression. Early preclinical studies suggest that combining sorafenib with HSP90 inhibitors can increase apoptosis in cancer cells and improve median survival in animal models [31]. Research also focuses on combining TKIs with other agents, like BRAF inhibitors (e.g., dabrafenib) and mitogen-activated protein kinase (MEK) inhibitors (e.g., trametinib), particularly for patients with BRAF mutations. These combinations have shown enhanced therapeutic effects and may offer a more effective treatment strategy for advanced thyroid cancers [32]. The treatment landscape for FTC is evolving with these targeted therapies and combination strategies. While TKIs such as lenvatinib and sorafenib offer significant benefits, ongoing research into combination therapies aims to improve outcomes and address resistance and side effect challenges. Continued investigation is essential for optimizing patient care in FTC [32]. Treatment options for FTC are shown in Figure 1.

Role of External Beam Radiation Therapy (EBRT)

EBRT is an important treatment modality for FTC, particularly in specific clinical contexts [33]. EBRT is often used as an adjuvant therapy following surgery, mainly when the tumor exhibits aggressive features, has spread to adjacent lymph nodes, or presents a high risk of recurrence. In such cases, EBRT helps eliminate residual cancer cells and reduces the likelihood of cancer returning. Additionally, EBRT is valuable in palliative care, providing symptom relief, such as alleviating pain caused by tumor growth or metastasis, thereby significantly enhancing the quality of life for patients with advanced disease [33]. For patients with unresectable tumors, where surgical removal is not feasible due to the tumor's size or location, EBRT can serve as a primary treatment to control tumor growth and manage disease progression. In some cases, EBRT may also be administered preoperatively to shrink tumors, making them easier to remove surgically. It is also beneficial for patients with recurrent cancer or those with specific tumor types susceptible to radiation, providing a crucial tool in the oncologist's treatment arsenal [34]. Modern techniques, such as intensity-modulated radiation therapy (IMRT) and volumetric arc therapy (VMAT), have significantly enhanced the effectiveness of EBRT in treating FTC [35]. These advanced methods allow for exact radiation delivery, minimizing damage to surrounding healthy tissues and reducing side effects. Consequently, patients receiving EBRT as part of their treatment regimen often experience better outcomes compared to those who do not receive radiation therapy. In palliative settings, EBRT has proven effective in alleviating pain and improving overall quality of life, demonstrating its value beyond curative treatment [35]. Ongoing research aims to refine the application of EBRT in FTC management, with advancements in radiation technology focused on optimizing treatment protocols and improving patient outcomes. As our understanding of FTC evolves, the role of EBRT is expected to expand, further solidifying its importance in the comprehensive management of FTC [36].

Post-treatment management

Surveillance Strategies (Tg Levels and Imaging)

After treatment, surveillance strategies for FTC primarily involve monitoring serum Tg levels and employing imaging techniques. These approaches are crucial for detecting recurrence and effectively managing patient outcomes [37]. Serum Tg levels are a crucial biomarker used to monitor patients following treatment for DTC, including FTC. After total thyroidectomy and RAI ablation, Tg levels indicate the presence of any remaining thyroid tissue, whether benign or malignant. The highest sensitivity and specificity for detecting recurrent disease are achieved when Tg levels are measured following these initial treatments [38]. Tg measurements can be performed under two conditions: thyroid-stimulating hormone (TSH)-suppressed and TSH-stimulated. In the TSH-suppressed state, patients receive suppressive doses of thyroid hormone to keep TSH levels low, a method commonly used for low- and intermediate-risk patients. TSH-stimulated measurements involve either withdrawing thyroid hormone or administering recombinant human TSH (rhTSH) to elevated TSH levels. While the latter approach increases sensitivity for detecting recurrence, it is less commonly employed for low-risk patients, as the added sensitivity does not significantly impact management decisions. Additionally, measuring anti-thyroglobulin antibodies (anti-Tg Ab) is important, as these antibodies can interfere with Tg measurements. Monitoring these antibodies alongside Tg levels provides a more accurate assessment of disease status [39]. Neck ultrasound is a valuable tool in FTC surveillance, particularly for detecting structural changes in the thyroid bed and identifying lymph node metastases. Ultrasound often reveals lesions that may be too small for other imaging modalities, making it a preferred method for routine follow-up. In cases of significant Tg level increases or concerning clinical findings, additional imaging techniques, such as CT or MRI, may be used. These modalities help localize recurrent disease and assess the extent of metastasis [40]. Combining serum Tg monitoring and imaging studies is essential for post-treatment surveillance of FTC. Regular assessments enable timely detection of recurrence, guiding further management and improving patient outcomes. These strategies should be tailored to the individual risk profile, balancing the need for vigilance with the potential for unnecessary interventions [41].

Management of Recurrent or Metastatic Disease

Management of recurrent or metastatic FTC requires a comprehensive approach tailored to the individual patient’s disease characteristics and treatment history. Surgical options, such as salvage surgery, may be considered for localized recurrences where complete resection is achievable; however, many patients present with advanced disease that is unsuitable for further surgical intervention [42]. RAI therapy is a cornerstone in managing DTCs, including FTC. It is typically administered postoperatively to ablate residual thyroid tissue and treat known metastases. Nonetheless, a significant proportion of patients (25-50%) may become RAI-refractory, meaning their tumors do not effectively uptake iodine, which limits the efficacy of this treatment. For patients who initially respond to RAI but later exhibit signs of recurrence, repeat RAI may be necessary. Monitoring Tg levels is crucial for guiding the timing and need for additional RAI treatments [43]. In RAI-refractory cases, targeted therapies have demonstrated effectiveness, particularly VEGF receptor-targeted TKIs, such as lenvatinib and sorafenib. These agents can help control disease progression and improve patient outcomes, although they are associated with significant toxicity and side effects that require careful management. The initiation of these therapies is often deferred until clear evidence of disease progression is present to minimize potential adverse effects. While cytotoxic drugs have limited efficacy and are generally not preferred as first-line treatments for recurrent or metastatic FTC, they may be considered for select patients with progressive disease resistant to other therapies [44]. When curative treatment is not feasible, palliative care becomes a critical management aspect. This involves symptom management, addressing treatment-related side effects, and providing psychological support to enhance the quality of life for patients with advanced disease [45]. Effective management of recurrent or metastatic FTC typically involves a multidisciplinary team, including oncologists, surgeons, radiologists, and supportive care specialists, ensuring comprehensive care that addresses medical and psychosocial needs [45].

Long-Term Outcomes and Quality of Life

Long-term outcomes and quality of life for patients with FTC have received considerable attention in recent years, demonstrating a generally favorable prognosis while also highlighting various treatment and survivorship challenges [46]. Research indicates that long-term survival rates for FTC patients are promising, with cumulative incidence rates of all-cause mortality approximately 24% and 45% at 10 and 20 years, respectively. A comprehensive analysis involving 1,167 patients with DTC, including FTC, reported a 10-year overall survival rate of around 92.5%. These statistics reflect the effectiveness of current treatment strategies for FTC, though recurrence remains a significant concern, especially for patients with high-risk features [47]. Recurrence rates in FTC can vary based on several prognostic factors. Key indicators influencing long-term outcomes include age at diagnosis, tumor size, and distant metastases. For example, patients diagnosed at an older age (over 60 years) or those with larger tumors (greater than 4 cm) generally have poorer prognoses [48]. Lymph node metastases are also associated with an increased risk of FTC-specific mortality. These factors highlight the importance of personalized treatment plans and diligent follow-up care to monitor for potential recurrence [48]. The impact of treatment on quality of life is another critical aspect of long-term management for FTC patients. While surgical interventions and RAI therapy are effective in controlling the disease, they can lead to long-term side effects, such as hypothyroidism [49]. This condition requires lifelong thyroid hormone replacement therapy, which can affect metabolic health and overall well-being. Additionally, the psychological impact of a cancer diagnosis can significantly influence quality of life, with many patients experiencing anxiety and depression related to their diagnosis and the ongoing fear of recurrence. Psychological support is, therefore, a crucial component of post-treatment care [49]. To address these challenges, regular follow-up care is essential for monitoring potential recurrences and managing the long-term effects of treatment. This includes routine blood tests to assess Tg levels and imaging studies as needed [50]. Effective communication with healthcare providers about symptoms and concerns can help alleviate the psychological burden associated with cancer survivorship. Overall, while the long-term outcomes for FTC patients are generally positive, ongoing support and monitoring are vital to enhance their quality of life and ensure optimal health management [50].

Emerging therapies and future directions

The treatment landscape for FTC is rapidly evolving due to advancements in molecular biology and the introduction of novel therapeutic agents. In recent years, several TKIs have been approved for advanced, radioiodine-refractory DTCs, including FTC [51]. Notable TKIs, such as lapatinib and sorafenib, have become standard first-line treatments for locally advanced or metastatic FTC that do not respond to RAI therapy. These multikinase inhibitors target multiple pathways involved in tumor growth and angiogenesis, demonstrating significant improvements in PFS rates. Furthermore, with the identification of rearranged during transfection (RET) mutations in some FTC cases, selective RET inhibitors are being explored as potential treatment options, personalizing therapy based on genetic profiling [51]. Immunotherapy is a promising approach for FTC, particularly for patients with advanced disease. Checkpoint inhibitors targeting the PD-1/PD-L1 axis have shown potential in reducing tumor growth in preclinical models. Combining these inhibitors with TKIs may enhance therapeutic efficacy by modulating the tumor microenvironment and improving immune responses [52]. Additionally, chimeric antigen receptor (CAR) therapies are being investigated for liquid and solid tumors, including FTC. These therapies aim to harness the immune system to target and destroy cancer cells, representing a novel treatment avenue [52]. Looking forward, the future of FTC treatment will likely hinge on integrating molecular profiling and personalized medicine. Key focus areas include developing biomarkers to predict responses to specific therapies, enhancing patient selection for targeted treatments, and potentially leading to improved outcomes. Exploring combinations of TKIs and immunotherapies may provide synergistic effects, improving overall response rates and the durability of treatment responses. Ongoing clinical trials are essential for evaluating new agents and combinations and understanding their long-term effects and potential toxicities [53]. Additionally, research into the tumor microenvironment's role in FTC progression and treatment resistance will inform future therapeutic strategies. A deeper understanding of how the microenvironment influences tumor behavior could lead to novel interventions that modify the immune landscape of tumors, ultimately improving treatment efficacy [54].

Challenges and controversies

FTC presents several challenges and controversies in its diagnosis and management, primarily due to diagnostic variability, treatment approaches, and the evolving understanding of its biological behavior [55]. One significant challenge in diagnosing FTC lies in the interobserver variability among pathologists. Studies have shown that consensus diagnoses occur in only a minority of cases, leading to discrepancies in the classification of thyroid lesions. Factors contributing to this variability include the degree of nuclear atypia and the interpretation of capsular or vascular invasion, which are critical for distinguishing FTC from other entities, such as follicular adenoma and the follicular variant of PTC. Due to overlapping cytological features, fine-needle aspiration cytology (FNAC) often fails to provide definitive diagnoses for follicular neoplasms [56]. The diagnostic hallmarks of FTC, such as vascular and capsular invasion, cannot be reliably assessed through cytology alone, necessitating histological verification post-surgery. This limitation results in a low positive predictive value, with only about 20% of cytologically diagnosed follicular neoplasms ultimately confirmed as carcinoma. The classification of FTC has evolved, with recent changes in understanding its histological characteristics. For instance, the introduction of non-invasive follicular thyroid neoplasm with papillary-like nuclear features (NIFTP) as a benign entity has further complicated the diagnostic landscape, reclassifying previously diagnosed cases and impacting survival statistics [57]. The standard treatment for FTC typically involves total thyroidectomy followed by RAI therapy. However, there is ongoing debate regarding the management of MI-FTC, which may not require the completion of thyroidectomy [58]. Some studies have reported cases of distant metastasis and recurrence even in patients classified as having minimally invasive disease, raising questions about the adequacy of initial surgical approaches. The advent of targeted therapies, such as TKIs (e.g., lenvatinib and vandetanib), has changed the treatment landscape for advanced FTC. While these therapies show promise, their optimal use, particularly in patient selection and timing, remains a subject of investigation. The integration of these novel agents into standard treatment protocols is still evolving, leading to uncertainty in clinical practice [58]. The challenges and controversies surrounding FTC underscore the need for ongoing research to refine diagnostic criteria and treatment protocols. Enhanced molecular profiling and the development of more precise diagnostic tools, such as gene expression analysis, may improve preoperative differentiation between benign and malignant lesions [59]. Furthermore, understanding the molecular underpinnings of FTC could lead to more targeted and effective therapies, ultimately improving patient outcomes. In summary, the complexities associated with the diagnosis and management of FTC necessitate a multidisciplinary approach and continued dialogue within the medical community to address these ongoing challenges [59].

## Conclusions

In conclusion, advancements in the diagnosis and treatment of FTC have significantly enhanced our ability to manage this complex malignancy effectively. Integrating advanced imaging techniques, improved biopsy methods, and molecular diagnostics has led to more accurate and timely diagnoses, which are crucial for devising appropriate treatment plans. Current therapeutic approaches, including surgical interventions, RAI therapy, and targeted treatments, have demonstrated varying effectiveness, offering hope for improved patient outcomes. Despite these advancements, challenges remain, including variability in treatment guidelines and disparities in access to care. Looking ahead, ongoing research and emerging therapies hold promise for further improving the management of FTC. Continued exploration of novel diagnostic tools and therapeutic strategies will be essential in refining treatment approaches and addressing existing gaps. A comprehensive understanding of FTC and its evolving management strategies will ultimately contribute to better patient care and outcomes, underscoring the importance of continued research and innovation in this field.
